# An Integrated Strategy for Preventing and Rehabilitating Dust-Induced Occupational Bronchopulmonary Diseases: A Scoping Review

**DOI:** 10.3390/arm93040030

**Published:** 2025-08-13

**Authors:** Alexandr E. Gulyayev, Karlygash S. Absattarova, Sayagul A. Kairgeldina, Raushan S. Dosmagambetova, Kanat K. Tekebayev, Madina B. Baurzhan, Nazym Sagandykova, Gaukhar Sh. Dauletova

**Affiliations:** 1 Scientific Research Department, Ministry of Health of the Republic of Kazakhstan, Research Institute of Balneology and Medical Rehabilitation, Astana 010000, Kazakhstan; akin@mail.ru (A.E.G.); karlygash_absatt@mail.ru (K.S.A.); sanborovoe@mail.kz (S.A.K.); dosmagambetovaraushan@gmail.com (R.S.D.); kanat_7@mail.ru (K.K.T.); 2 Laboratory of Drug Discovery and Development, Nazarbayev University, Astana 010000, Kazakhstan; 3 Department of Medical Rehabilitation and Sports Medicine, NJSC Astana Medical University, Astana 010000, Kazakhstan; dauletova.g@amu.kz; 4 Internal Diseases Department, Corporate Fund “University Medical Center”, Astana 010000, Kazakhstan; doctor.ent.alm@gmail.com

**Keywords:** chronic obstructive pulmonary disease, prevention, scoping review

## Abstract

**Highlights:**

**What are the main findings?**

- Dust-induced occupational bronchopulmonary diseases—often overlooked contributors to the global COPD burden—represent a significant cause of preventable morbidity and mortality among exposed workers.
- Dust-induced occupational bronchopulmonary disease is a common condition in patients with COPD, often requiring increased energy, protein, vitamin, and mineral requirements.

**What is the implication of the main finding?**

- Preventing COPD and OBPDs requires accurate screening using both clinical tools (e.g., HRCT, FeNO) and biological markers (e.g., IL-6, TNF-α).
- Integrating personalized nutritional support and antioxidant-based rehabilitation—such as polyphenol-rich phytotherapy—can reduce progression and improve work reintegration outcomes.
- Occupational health systems should prioritize early detection programs and structured rehabilitation tailored to dust-exposed populations.

**Abstract:**

Background: Occupational bronchopulmonary diseases (OBPDs)—including pneumoconiosis, silicosis, and occupational COPD—remain a pressing public health issue, especially in regions with intensive mining, metallurgy, and construction industries. Caused by chronic inhalation of fibrogenic dusts, these conditions are often diagnosed at late stages, resulting in irreversible lung damage and diminished work capacity. Methods: A scoping review was performed using the Arksey and O’Malley framework, with methodological refinements from the Joanna Briggs Institute. Following PRISMA-ScR guidelines, we searched PubMed, Scopus, and gray literature for publications from 2014 to 2024. After screening 1761 records and full-text review, nine studies were included in the final synthesis, comprising two systematic reviews, two narrative literature reviews, and five observational studies. Results: Key risk factors identified included prolonged exposure to silica and coal dust, tobacco use, and genetic susceptibility. Diagnostic delays were attributed to the underuse of high-resolution CT and exhaled nitric oxide analysis. Several studies highlighted the diagnostic value of oxidative stress and inflammatory markers (e.g., IL-6, TNF-α). Nutritional rehabilitation and polyphenol-enriched herbal therapies were associated with improved respiratory function and quality of life. However, these strategies remain underutilized, particularly in low-resource settings. Conclusions: A coordinated, biomarker-driven approach integrating early diagnosis, dust exposure control, and tailored rehabilitation is urgently needed. Multidisciplinary models may reduce the clinical and socioeconomic burden of OBPDs.

## 1. Introduction

Occupational bronchopulmonary diseases (OBPDs) represent a class of chronic respiratory conditions that are directly linked to prolonged exposure to harmful aerosols in industrial environments. Among these, dust-induced lung diseases—including pneumoconiosis, chronic obstructive pulmonary disease (COPD), and silicosis—remain some of the most prevalent and debilitating, especially in sectors like mining, metallurgy, and coal production. Despite the evolution of protective regulations, these diseases continue to impact thousands of workers globally each year [1,2].

The burden of OBPDs is particularly pronounced in low- and middle-income countries where industrial hygiene standards are inconsistently enforced and access to early diagnostics and medical care is limited [3]. In Kazakhstan, for instance, regions with high concentrations of extractive industries report alarming rates of occupational lung pathologies. Industrial dust, particularly crystalline silica and coal dust, can cause fibrotic changes in the lungs that are progressive and irreversible, even after exposure cessation [4]. According to the Global Burden of Disease (GBD) Study 2016, occupational exposure to particulate matter, gases, and fumes (PMGF) was among the leading contributors to disability-adjusted life years (DALYs), accounting for over 76 million DALYs globally (~3.2% of total DALYs). The WHO/ILO Joint Estimates further specify that in 2016, approximately 450,000 deaths were attributable to PMGF exposure and nearly 745,000 deaths were linked to long working hours, underscoring occupational respiratory risks as a major health concern. The International Labour Organization (ILO) also reports that occupational respiratory diseases represent around one-third of all registered work-related illnesses and contribute to 10–20% of occupational fatalities globally [5,6].

What makes these conditions especially challenging is their insidious onset. Many dust-induced diseases develop gradually and remain asymptomatic or present only mild symptoms until reaching advanced stages, by which time significant pulmonary damage has often occurred. Consequently, early detection remains a critical yet underutilized tool in occupational health management. Numerous studies have highlighted emerging diagnostic techniques such as high-resolution computed tomography (HRCT), fractional exhaled nitric oxide (FeNO), and the use of oxidative stress and inflammatory biomarkers. However, integration of such tools into routine screening and personalized rehabilitation strategies remains limited. Rehabilitation models for OBPDs are still evolving and often lack multidisciplinary coordination, especially in resource-limited settings.

Moreover, the economic repercussions of occupational lung diseases are profound. Affected workers may face permanent disability, job loss, and reduced life expectancy, which in turn lead to increased pressure on healthcare systems, loss of productivity, and escalating compensation costs for employers and social insurance programs [7]. According to recent studies, the indirect costs of long-term illness, such as early retirement and family caregiving, often exceed direct treatment expenditures [8].

In response to these complex and layered challenges, international public health experts are calling for integrated strategies that address the full spectrum of dust-induced OBPDs, from primary prevention and early screening to comprehensive rehabilitation [9]. Prevention must be proactive, involving environmental controls, personal protective equipment (PPE), and routine surveillance. Yet, it must also be biologically informed: new research indicates that genetic polymorphisms, oxidative stress markers, and immune parameters may help identify individuals at higher risk [10].

Sanatorium–resort treatment and rehabilitation programs also play a pivotal role in restoring pulmonary function and enhancing quality of life. Modern rehabilitation should not be limited to traditional physiotherapy, but include personalized protocols based on biomarker analysis, respiratory function tests, and immune profiling [11,12]. These strategies must be adaptive to specific occupational contexts and population genetics, especially in countries where occupational exposures differ significantly from international norms.

Furthermore, an effective national strategy should harmonize efforts between regulatory institutions, occupational physicians, employers, and workers. A systemic approach could include mandatory health screening for high-risk employees, digital monitoring of aerosol concentrations in workplaces, and government-supported rehabilitation for diagnosed cases. This would align with the broader global movement toward preventive occupational medicine and evidence-based public health.

In summary, despite the long-standing awareness of the risks posed by industrial dust, dust-induced bronchopulmonary diseases remain a neglected area of occupational health in many countries. A coordinated, scientifically grounded, and locally contextualized approach to both prevention and rehabilitation is urgently needed to reduce the burden of these diseases. This review aims to synthesize the current evidence base and provide a comprehensive map of available strategies, gaps in knowledge, and directions for future research and policy.

Given the heterogeneity of the existing research, this scoping review aims to synthesize and map the current evidence regarding the prevention, diagnosis, and rehabilitation of dust-induced OBPDs, with a specific emphasis on identifying gaps in implementation, highlighting effective interventions, and informing future research, clinical practice, and public health policy. By adopting a structured and comprehensive mapping approach, this review seeks to support decision-making and guide the development of integrated prevention and rehabilitation frameworks.

Ultimately, the findings of this review are intended to guide stakeholders in developing evidence-based, multidisciplinary, and scalable interventions for improving respiratory health among exposed worker populations.

## 2. Materials and Methods

### 2.1. Review Framework and Methodological Approach

This scoping review was conducted in accordance with the five-stage methodological framework originally outlined by Arksey and O’Malley [13], which includes the following steps: identifying the research question; identifying relevant studies; study selection; charting the data; and collating, summarizing, and reporting the results. These core stages were enhanced using the methodological guidance provided by the Joanna Briggs Institute (JBI) [14], which offers additional clarity on protocol design, eligibility criteria, and data presentation strategies. The Arksey and O’Malley framework was chosen for its suitability in mapping complex and heterogeneous evidence bases, particularly in fields with evolving research such as occupational lung disease. Its flexibility in incorporating diverse study designs and outcomes made it appropriate for capturing the breadth of OBPD research.

### 2.2. Reporting Standards

The review adhered to the PRISMA-ScR (Preferred Reporting Items for Systematic Reviews and Meta-Analyses extension for Scoping Reviews) guidelines [15]. This standard ensures completeness and transparency in the review process, including a detailed presentation of inclusion criteria, data sources, and synthesis methods. A review protocol was not registered in PROSPERO as scoping reviews without outcome-based intervention data are not eligible for registration on the platform. However, a structured methodology was predefined by the research team and adhered to throughout the review process. Searches were conducted in PubMed, Scopus, and gray literature sources, covering the period from January 2014 to January 2024.

### 2.3. Study Selection and Data Charting

Data extraction and charting were carried out using a standardized data charting form developed in Microsoft Excel. This form included fields for study characteristics (design, country, sample size), target population, intervention type, and main findings. To ensure consistency, two reviewers independently piloted the form on a subset of studies and resolved discrepancies through consensus.

#### Quality Appraisal of Included Studies

As this is a scoping review, formal critical appraisal of included studies was not a mandatory component. However, methodological rigor and thematic relevance were considered during study selection. Studies were screened for scientific validity based on clarity of design, relevance to occupational exposures, and data completeness. No standardized tools such as the JBI Critical Appraisal Checklist were applied, which constitutes a limitation discussed further below.

### 2.4. Synthesis of Results

Results were synthesized narratively. Due to the heterogeneity of the study designs, interventions, and outcomes, no meta-analysis was conducted. Instead, findings were categorized thematically into three domains: primary prevention; diagnostic and risk assessment methods; and rehabilitation and recovery strategies. Frequency counts and descriptive summaries were used to highlight patterns and gaps in the literature. A PRISMA-ScR checklist was followed during the review process to ensure transparency and completeness. Adherence was cross verified independently by two authors to minimize bias and improve methodological consistency.

## 3. Results

### 3.1. Study Selection

A total of 1784 records were identified through database and gray literature searches. After removal of 517 duplicates, 1267 titles and abstracts were screened. Based on predefined inclusion criteria, 1228 records were excluded. The remaining 39 full-text articles were assessed for eligibility, of which 30 did not meet the inclusion criteria. A total of nine studies were included in the final synthesis, comprising two systematic reviews, two narrative literature reviews, and five observational or descriptive studies. The distribution of focus areas across the included studies is summarized in Table 1.

The heterogeneity in sample sizes and geographic coverage highlights the variability in the exposure conditions and response strategies. For instance, Su et al. [16] synthesized data from multiple global cohorts, whereas Sim et al. [7] reported on a national-level registry of 263 confirmed silicosis cases in Australia. Conversely, several U.S.-based narrative reviews provided conceptual frameworks but lacked empirical population-level data [17,18,19,20].

Five studies primarily addressed diagnostic strategies and clinical criteria, including the application of HRCT, exhaled nitric oxide, and biomarker panels such as IL-6 and TNF-α [17,18,19,20,21].

Three studies focused on preventive and workplace control measures, including dust suppression protocols and occupational surveillance programs [1,7,22].

Two studies specifically explored rehabilitation approaches, such as nutrition-based therapy and return-to-work interventions [7,22].

Table 2 provides a consolidated overview of the design, population, objectives, and key outcomes of each study. While diagnostic strategies were covered in depth—including novel imaging and biomarker integration—none of the studies explicitly reported cost-effectiveness analyses or feasibility evaluations of these tools in real-world clinical settings.

Across the reviewed studies, diagnostic themes appeared more frequently than those addressing rehabilitation, indicating a potential research gap in post-diagnosis patient care models. This trend may reflect the relative complexity and underfunding of long-term occupational rehabilitation in low- and middle-income countries (LMICs).

**Figure 1:**
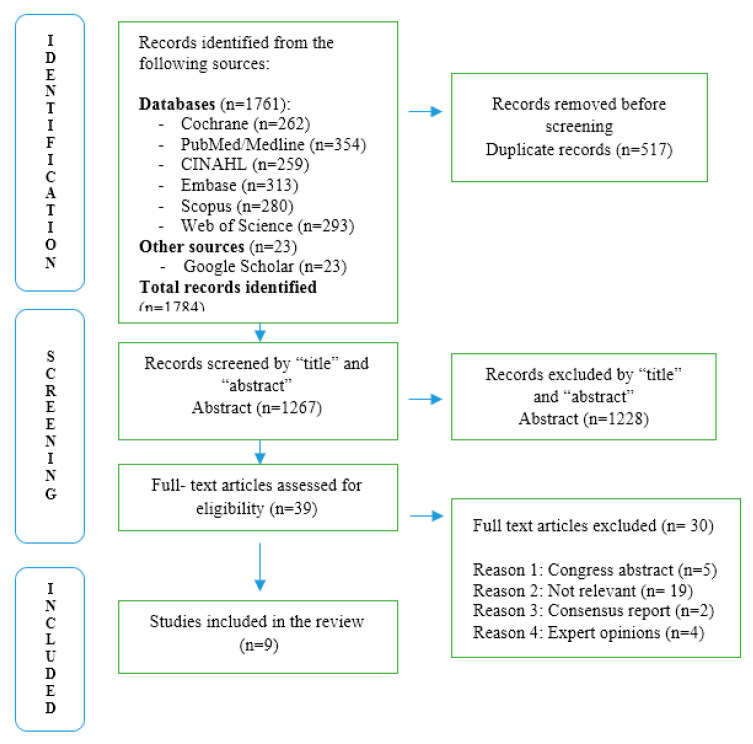
PRISMA-ScR flow chart of study selection process.

A visual representation of the selection process is provided in the PRISMA-ScR flow diagram in Figure 1, illustrating the selection process for studies included in the scoping review (n = 9) [13].

### 3.2. Characteristics of Included Studies

The selected studies span the years 2014 to 2024, and were conducted in countries including China, USA, UK, North Macedonia, and Australia.

The target populations were industrial workers exposed to coal dust, silica, or mixed aerosols, with a particular focus on silicosis, pneumoconiosis, and occupational COPD. While the primary population consisted of industrial workers exposed to respirable dusts (e.g., silica, coal), most studies did not provide detailed demographic breakdowns such as age, sex, or duration of occupational exposure. This lack of disaggregated reporting limits the ability to assess differential risks across population subgroups and should be addressed in future research.

#### 3.2.1. Eligibility Criteria

The following inclusion and exclusion criteria were applied during study selection (see Table 1):

**Table 1:** The focus areas addressed in the reviewed studies.

Criterion Type	Inclusion Criteria	Exclusion Criteria
Population	Workers exposed to occupational dusts (e.g., silica, coal) with diagnosis or risk of OBPDs	Studies focused on general population, non-occupational exposures, comorbidities unrelated to OBPD
Study design	Systematic reviews, narrative literature reviews, observational studies, descriptive studies	Randomized controlled trials unrelated to occupational or public health interventions
Publication type	Peer-reviewed articles, government reports, mechanistic analyses	Non-scientific articles, case reports, conference abstracts, editorials without primary data
Time frame	Publications from January 2014 to January 2024	Studies published outside this period
Language	English language publications	Non-English publications without accessible translation

We included studies focusing on occupational exposures to respirable dusts and related lung diseases. Excluded were reports focusing on general population cohorts, unrelated comorbidities, or purely methodological articles without empirical data.

Based on the eligibility criteria outlined above, an initial pool of 1761 records was screened by title and abstract. Following full-text review, nine studies were included in the final synthesis. These met the defined inclusion criteria for relevance, methodological rigor, and thematic focus on dust-induced occupational bronchopulmonary diseases (OBPDs).

#### 3.2.2. Excluded Studies

Studies were excluded primarily for the following reasons:

- Irrelevance to occupational exposure contexts (e.g., general pulmonary disease studies);
- Lack of empirical data or focus on unrelated health outcomes;
- Publications outside the 2014–2024 timeframe;
- Publications not written in English.

The PRISMA flow diagram (Figure 1) illustrates the selection process in detail. All selected studies aligned with the criteria for population, design, and intervention relevance as presented in Table 2.

### 3.3. Thematic Synthesis

#### 3.3.1. Risk Factors and Pathogenesis

Multiple studies highlighted prolonged exposure to silica and coal dust as major risk factors for occupational bronchopulmonary diseases (OBPDs). Su et al., 2023 [16] identified exposure duration, ambient dust concentration, and smoking as significant predictors of pneumoconiosis. Vanka et al., 2022 [17] and Weissman, 2022 [18] explored the molecular mechanisms underlying dust-induced lung fibrosis, emphasizing oxidative stress, macrophage activation, and pro-inflammatory cytokine pathways (e.g., IL-6, TNF-α, TGF-β).

#### 3.3.2. Diagnostics and Clinical Criteria

Updated clinical recommendations by Krefft et al., 2020 [19] stressed the importance of using high-resolution CT (HRCT) and gathering a thorough occupational history for early detection of silicosis. Perlman & Maier, 2019 [20] also underscored the value of spirometry, biomarker assays, and structured symptom tracking to differentiate OBPD from non-occupational respiratory conditions.

#### 3.3.3. Prevention and Surveillance

Cullinan et al., 2017 [1] and Minov et al., 2022 [21] reviewed preventive strategies for both traditional and emerging occupational exposures. Key measures included workplace dust monitoring, personal protective equipment (PPE) usage, smoking cessation programs, and regulatory surveillance. The need for integrated workplace health policies and routine spirometric screening was emphasized across multiple studies.

#### 3.3.4. Rehabilitation and Return to Work

Three studies focused on rehabilitation strategies. Hou X. et al., 2024 [22] and Sim et al., 2019 [7] demonstrated that structured rehabilitation programs, including sanatorium–resort treatment and vocational reintegration, led to improved outcomes in functional status and work re-entry. Sim et al. additionally reported gaps in existing return-to-work systems and advocated for personalized rehabilitation plans supported by multidisciplinary teams.

### 3.4. Overview Table

The included studies demonstrate that OBPDs are multifactorial diseases influenced by environmental, occupational, and host-related factors. A combined strategy of early diagnostics, risk-based monitoring, and rehabilitative care is critical to mitigating long-term disease burden. Table 2 provides a structured summary of the studies reviewed.

**Table 2:** General characteristics of the studies included.

Author (Year), Country	Research Design	Sample	Research Purpose	Intervention	Results
Cullinan et al. (2017), UK and EU [1]	Review with recommendations (belongs to observational studies)	Occupational exposure cases	Identify prevention strategies for both classic and emerging exposures	Epidemiological policy, recommendations	Suggested stronger regulations and surveillance in occupational medicine
Sim et al. (2019), Australia [7]	Government report (belongs to observational studies)	263 workers with silicosis	Explore rehabilitation and return-to-work models for silicosis patients	Vocational rehab program review	Identified need for customized return to work plans
Su et al. (2023), China [16]	Systematic review and meta-analysis	Multiple occupational cohorts	Assess incidence and risk factors of pneumoconiosis	Meta-analysis of global data	Identified key predictors including exposure duration, smoking, and silica concentration
Vanka et al. (2022), USA [17]	Mechanistic review (belongs to observational studies)	Preclinical and clinical	Explore dust-related immune and oxidative mechanisms	Mechanistic analysis (ROS/macrophages)	Identified fibrotic and inflammatory pathways in coal and silica exposure
Weissman (2022), USA [18]	Narrative review	Silicosis/CWP patients	Characterize progressive massive fibrosis (PMF)	Imaging and marker evaluation	Provided diagnostic criteria for early-stage PMF
Krefft et al. (2020), USA [19]	Clinical update (belongs to observational studies)	-	Update on clinical recognition and prevention of silicosis	Diagnostic algorithm and HRCT	Advocated regular screening and detailed exposure history
Perlman & Maier (2019), USA [20]	Narrative review		Summarize occupational lung disease management	Overview of therapies and policies	Stressed importance of early diagnosis and clinical monitoring
Minov et al. (2022), North Macedonia [21]	Review (belongs to observational studies)	COPD patient cohorts	Review COPD prevalence and prevention in occupational settings	Literature and guideline review	Highlighted need for dust control and cessation of smoking
Hou X. et al. (2025), China [22]	Systematic review	Coal-exposed populations	Assess health effects of coal dust and evaluate control interventions	Synthesis of environmental, clinical data	Proposed evidence-based dust suppression strategies for industry

## 4. Discussion

### 4.1. Diagnostics and Imaging Tools

This scoping review identified multiple layers of complexity in the prevention, diagnosis, and rehabilitation of dust-induced occupational bronchopulmonary diseases (OBPDs). These diseases, including pneumoconiosis, silicosis, and occupational COPD, develop gradually in individuals exposed to fibrogenic aerosols, such as coal dust and crystalline silica. Their clinical course is often insidious, with subclinical inflammation and irreversible fibrosis progressing before symptom onset.

Several studies emphasized the need for early detection. Diagnostic delays are largely attributed to nonspecific symptoms at early stages and the lack of screening protocols in many occupational sectors. As noted by Krefft et al. [19] and Perlman & Maier [20], reliance on conventional chest X-rays can miss early lesions, whereas high-resolution CT (HRCT), lung function tests, and non-invasive biomarkers such as exhaled nitric oxide (FeNO) show greater sensitivity in detecting pre-clinical pathology. While HRCT is sensitive to early fibrotic changes, its high cost and limited accessibility in low-resource settings hinder widespread adoption. None of the included studies offered formal cost–benefit analyses, indicating a gap in evaluating their feasibility in real-world settings.

### 4.2. Biomarkers and Genetic Factors

**Figure 2:**
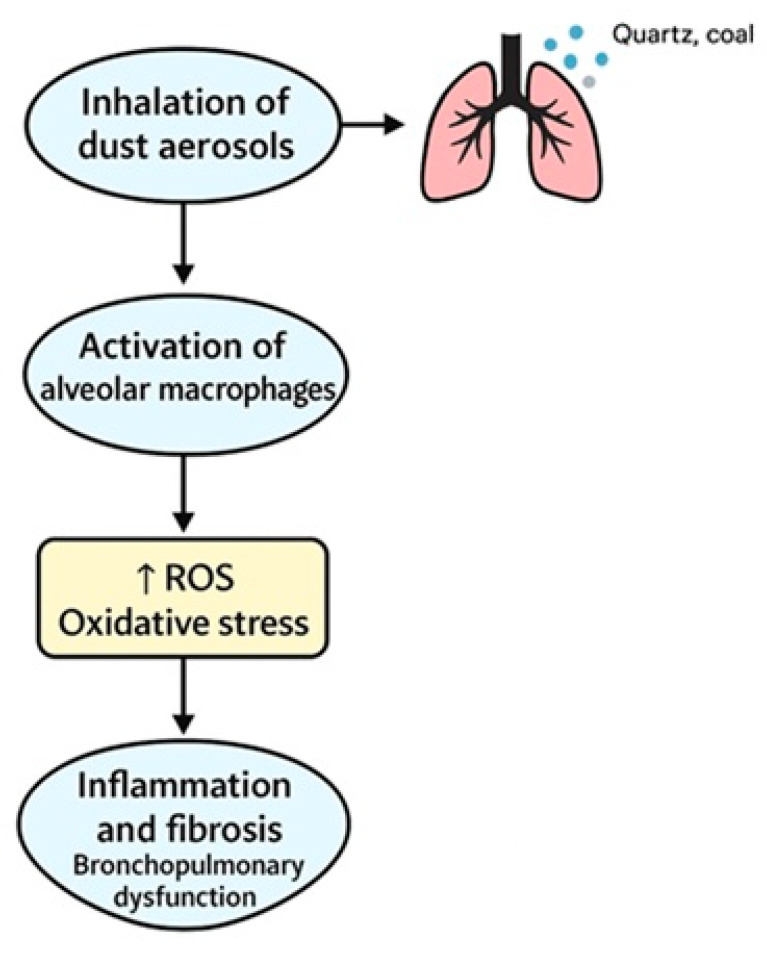
Pathogenetic mechanisms of dust-induced OBPDs. The figure illustrates the pathogenesis of bronchopulmonary dysfunction due to inhalation of dust aerosols such as quartz and coal. Particles entering the lungs activate alveolar macrophages, which leads to increased formation of reactive oxygen species (ROS) and, as a result, oxidative stress. The resulting stress triggers inflammation and fibrosis, which ultimately contributes to the development of respiratory dysfunction. The arrow ↑ indicates an increase in ROS levels.

Recent research highlights the potential of oxidative stress and immune system markers as prognostic tools. Elevated levels of interleukin-6 (IL-6), tumor necrosis factor alpha (TNF-α) [23,24,25], and other pro-inflammatory mediators have been detected in workers with prolonged exposure, suggesting a state of chronic immune activation. These findings support the use of cytokine profiling and oxidative stress indices in risk stratification, as also discussed in Vanka et al. [17] and Weissman [18,26,27]. However, most studies were observational, and no consensus exists on standardized reference levels or diagnostic thresholds. The role of genetic predisposition was noted in two studies, yet evidence remains preliminary and predominantly derived from small cohorts or animal models. Hence, genetic screening is not currently viable for clinical implementation. Figure 2 illustrates the main biological pathways involved in the onset and progression of occupational bronchopulmonary diseases caused by industrial dust exposure. It was inspired by the conceptual frameworks proposed in Vanka et al. (2022) [28] and Weissman (2022) [18], and includes oxidative stress, immune activation, and fibrotic remodeling [28,29].

An emerging area of focus is the role of genetic predisposition. While current literature remains sparse, studies suggest that genetic polymorphisms in inflammation and detoxification genes (e.g., TNF, GST, Nrf2 pathways) may influence individual sensitivity to dust-related injury [30]. In addition to widely studied biomarkers such as IL-6 and TNF-α, recent clinical studies have explored the use of serum soluble ST2 to predict pulmonary stress responses in high-performance environments [31]. Although primarily validated in sports cardiology, such biomarkers may have translational value for occupational health surveillance in at-risk workers [32]. Future research is warranted to develop precision-prevention frameworks based on gene–environment interaction models.

### 4.3. Preventive and Workplace Interventions

In terms of interventions, environmental controls and personal protective equipment (PPE) remain foundational. However, disparities in implementation persist globally. Cullinan et al. [1] advocated for stronger workplace policies, mandatory health surveillance, and cross-sectoral regulation to reduce the disease burden. Yet these systems are often underdeveloped in regions with heavy industrial labor and limited occupational health infrastructure. In many LMICs, enforcement of occupational safety regulations remains inconsistent, exacerbating disparities in worker protection. Interventional trials evaluating policy or PPE effectiveness were notably absent, limiting the ability to draw causal conclusions about intervention outcomes.

### 4.4. Rehabilitation and Multidisciplinary Care

**Figure 3:**
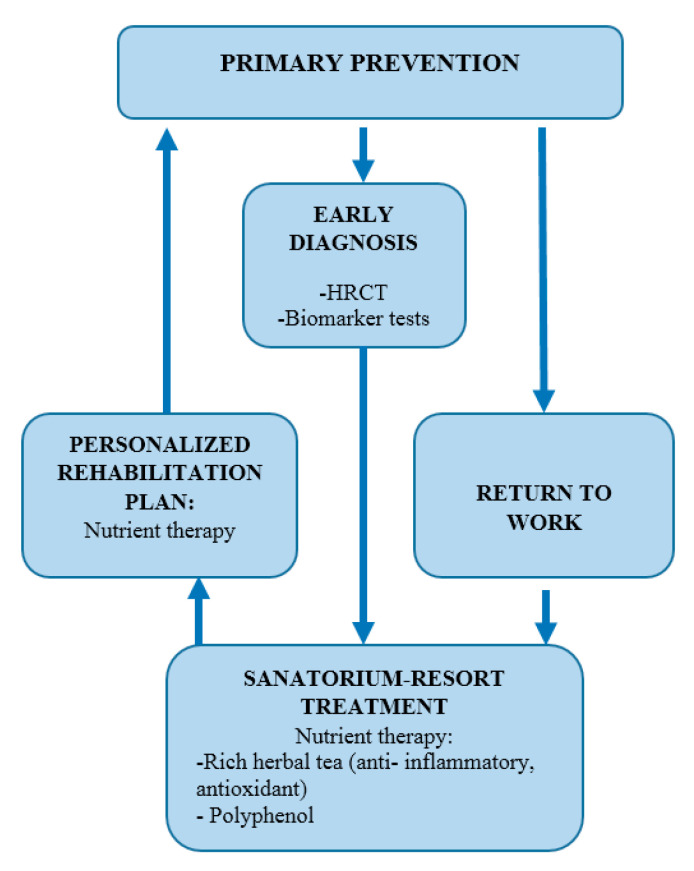
Multilevel strategy for prevention and rehabilitation.

Rehabilitation is another critical, yet often under-addressed, component. Sim et al. [7] and Hou X. et al. [22] provided evidence for the benefits of structured sanatorium–resort rehabilitation, combining physiotherapy, immune-modulating interventions, and nutritional support. These programs not only improve lung function but also reduce the psychosocial burden of chronic disease and facilitate return to work. Only a minority of studies examined rehabilitation interventions despite evidence of their clinical benefits. The underrepresentation of rehabilitation reflects a broader issue: post-diagnosis management is often deprioritized in occupational health systems, especially in resource-limited contexts. Nevertheless, many countries lack standardized rehabilitation pathways, and multidisciplinary collaboration remains insufficient. A schematic model depicting an integrated approach to managing dust-induced occupational lung diseases is illustrated in Figure 3. It includes workplace controls, health surveillance, biomarker-guided screening, and personalized pulmonary rehabilitation. The conceptual structure was adapted from Sim et al. [7] and expanded using the synthesis from the current review.

Importantly, the findings of this review reinforce the need for a biopsychosocial model of care, incorporating biological risk profiling, workplace adaptation, and reintegration support to holistically manage OBPDs. This review adopted a scoping methodology due to the significant heterogeneity across study designs, populations, and outcome measures. As such, a meta-analysis was not attempted. This approach allowed for a broader mapping of available evidence and helped identify priority gaps for future research.

### 4.5. Methodological and Geographic Limitations

Most studies originated from high-income countries (HICs), particularly the USA, China, and Australia, with limited representation from Central Asia, Sub-Saharan Africa, or Latin America. The lack of geographic diversity may limit the applicability of findings to regions with different occupational exposures and health system capacities.

### 4.6. Justification for Scoping Review Approach

A meta-analysis was not performed due to the significant heterogeneity in study designs, outcome measures, and population characteristics across the included studies. The scoping review methodology was chosen to map the breadth of available evidence and identify critical gaps for future research.

### 4.7. Strengths

One of the primary strengths of this scoping review lies in its comprehensive and multidisciplinary approach to understanding dust-induced occupational bronchopulmonary diseases (OBPDs). By synthesizing literature from multiple regions and disciplines, the review maps current knowledge on prevention, early diagnostics, and rehabilitation—domains that are often studied in isolation.

Unlike traditional systematic reviews that may restrict inclusion based on narrow criteria, this review followed the Arksey and O’Malley framework and PRISMA-ScR guidelines, allowing for a broader exploration of the evidence, including that found in clinical trials, government reports, and mechanistic reviews. This inclusiveness improves external validity and policy relevance, especially for countries seeking integrated models of occupational health.

Additionally, by highlighting emerging areas, such as biomarker-based diagnostics, genetic susceptibility, and structured return-to-work models, the review identifies promising avenues for future translational research and health system planning.

### 4.8. Limitations

This review has several limitations. First, the absence of a quantitative synthesis limits the ability to calculate pooled effect sizes or conduct formal comparisons between interventions. The decision to not attempt a quantitative meta-analysis in this review aligns with the methodological framework of scoping reviews, which aim to map the existing evidence rather than synthesize effect sizes. Given the heterogeneity in study designs, populations, and outcomes among the included sources, statistical pooling was deemed inappropriate.

Second, despite efforts to include gray literature, certain industry- or government-led interventions may remain unpublished or inaccessible, particularly in low-resource settings. As a result, some effective but non-peer-reviewed programs may be underrepresented.

Third, while several studies suggest that genetic, immunologic, and molecular mechanisms may influence individual susceptibility to OBPDs, none of the included studies were designed as interventional trials directly targeting these biomarkers. As a result, while the potential for biologically tailored prevention appears promising, further prospective studies are needed to validate its clinical utility. These limitations affect the generalizability of findings and underscore the need for cautious interpretation when applying these insights across diverse occupational and geographic contexts.

## 5. Conclusions

This review reaffirms that dust-induced occupational bronchopulmonary diseases (OBPDs) represent a persistent and underrecognized global health challenge, particularly in sectors with high exposure to silica and coal dust. Despite notable advancements in diagnostic imaging and risk assessment, delayed recognition remains common, often leading to irreversible pulmonary impairment and significant socioeconomic consequences.

Our synthesis highlights the urgent need for an integrated, proactive approach to prevention and care. Evidence-based strategies should encompass the following elements:

- Early diagnostic interventions using HRCT, FeNO, and cytokine assays;
- Systematic workplace exposure surveillance and enforcement of dust control measures;
- Genetic and immunological risk profiling among vulnerable worker groups;
- Comprehensive worker education, including smoking cessation programs and occupational health literacy.

On the therapeutic front, personalized, multidisciplinary sanatorium–resort rehabilitation programs show promising outcomes in improving lung function, reducing psychosocial stress, and facilitating return-to-work pathways. Such interventions should be context-sensitive and adapted to national occupational health systems.

Sustainable progress in reducing OBPD incidence and disease progression will require close collaboration between public health institutions, employers, clinical practitioners, and researchers. Priority areas for future research include the following:

- Validation of biomarker-based screening for early disease detection;
- Cost-effectiveness analysis of screening and rehabilitation models;
- Development and deployment of digital platforms for exposure tracking and clinical decision support.

A coordinated, multidisciplinary response that is rooted in scientific evidence and tailored to occupational realities is essential to protect respiratory health, maintain workforce productivity, and minimize disability burdens among exposed populations.

## Data Availability

No new data were created or analyzed in this study.

## Abbreviations

The following abbreviations are used in this manuscript:

OBPD	Occupational bronchopulmonary diseases
COPD	Chronic obstructive pulmonary disease
PPE	Personal protective equipment
ScR	Scoping reviews
IL-6	Interleukin-6
TNF-α	Tumor necrosis factor alpha
TGF-β	Transforming growth factor beta
HRCT	High-resolution computed tomography
FeNO	Fractional exhaled nitric oxide
GST	Glutathione S-transferase
Nrf2	Nuclear factor erythroid 2–related factor 2
ROS	Reactive oxygen species
